# PERSON-CENTERED CARE PLANNING FOR PERSONS WITH MCC: LESSONS FROM PARTNER ROUNDTABLE DISCUSSIONS

**DOI:** 10.1093/geroni/igae098.1861

**Published:** 2024-12-31

**Authors:** Anne King, Sarina Schrager, Christie Jackson, LeAnn Michaels, David Dorr

**Affiliations:** Comagine Health, Portland, Oregon, United States; University of Wisconsin Madison, Madison, Wisconsin, United States; Oregon Health & Science University, Portland, Oregon, United States; Oregon Health & Science University, Portland, Oregon, United States; Oregon Health & Science University, Portland, Oregon, United States

## Abstract

The Partner Roundtable utilizes successive discussions with policymakers and health care leaders to identify barriers, facilitators, and strategies to improve person-centered care planning (PCCP) design and implementation in health systems, including primary and specialty care. The objectives are to: (1) identify organizational, policy, payment, technology, cost/resource, research, or other requirements for development and implementation of PCCP models across diverse health systems and settings, highlighting specific requirements, for persons living with, or at risk for, MCC; (2) identify challenges to implementation of PCCP; (3) recommend proven, potential, or innovative solutions or facilitators for implementation challenges; and (4) assist in dissemination of project findings. The Roundtable includes a diverse group of 25-30 individuals with influential leadership roles in making, shaping, and informing decisions related to PCCP implementation. Participants are decision-makers from health systems; local, state and federal health policy; health information technology; quality improvement and quality measurement; research; and patient associations or care recipients including family members or caregivers. Roundtable participants are representative of U.S. healthcare, including geographic spread, community health center leadership, payers, clinicians, and implementation and evaluation of innovative care models. Federal partners focused on improving care delivery for this population including care planning are invited participants. Roundtable expertise and recommendations, including environmental scan information, will inform future recommendations for promising models of PCCP.

